# Supplementary material for: Akt, IL-4, and STAT Proteins Play Distinct Roles in Prostaglandin Production in Human Follicular Dendritic Cell-like Cells
Source: Int J Mol Sci. 2023 Nov 24;24(23):16692. doi: 10.3390/ijms242316692 (PMC10706142; doi:10.3390/ijms242316692)
Supplement: Supplementary file 1 [file ijms-24-16692-s001.zip › ijms-2675727-supplementary.pptx]

## Slide 1
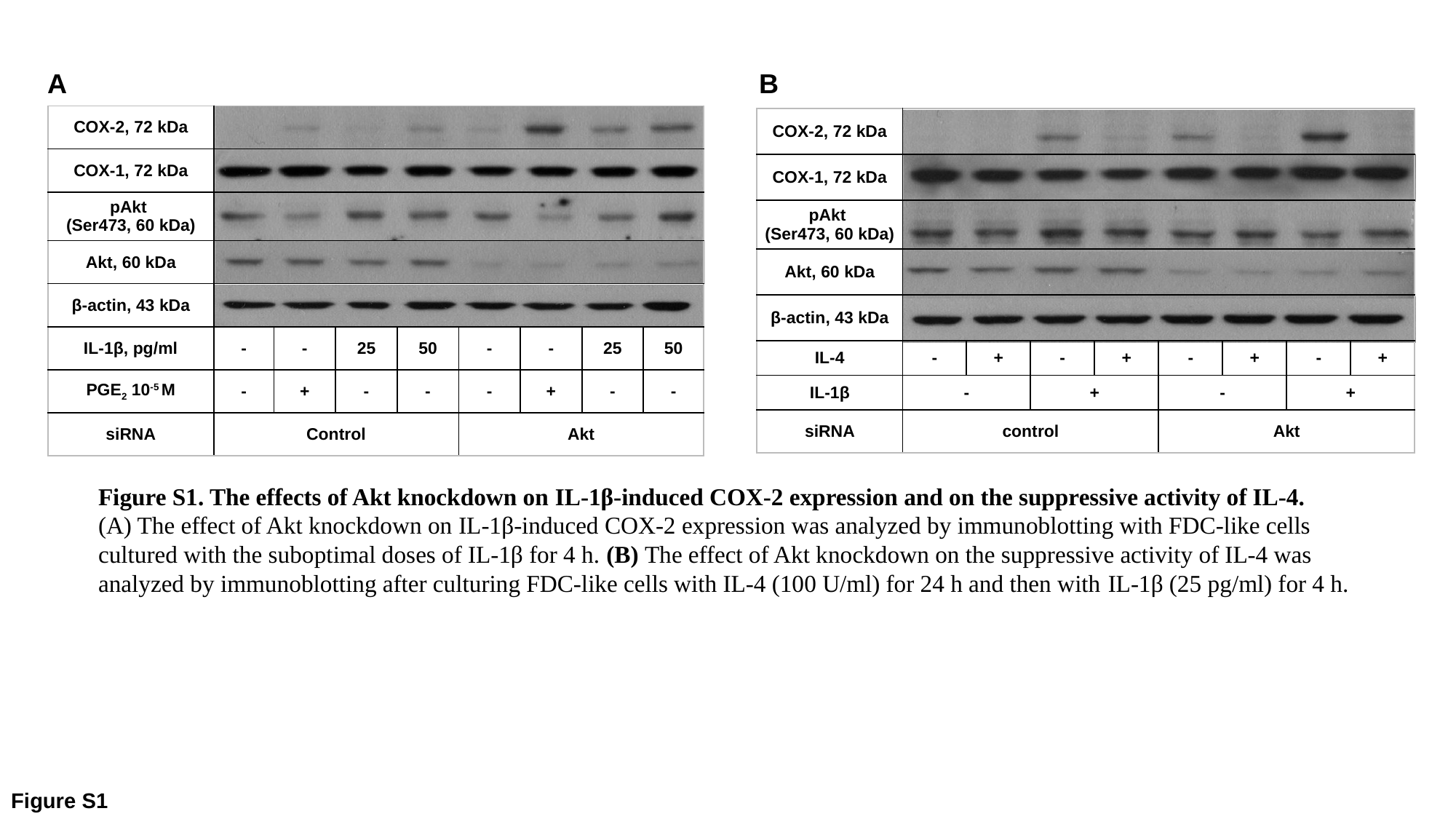

A B
| COX-2, 72 kDa | | | | | | | | |
| --- | --- | --- | --- | --- | --- | --- | --- | --- |
| COX-1, 72 kDa | | | | | | | | |
| pAkt (Ser473, 60 kDa) | | | | | | | | |
| Akt, 60 kDa | | | | | | | | |
| β-actin, 43 kDa | | | | | | | | |
| IL-1β, pg/ml | - | - | 25 | 50 | - | - | 25 | 50 |
| PGE2 10-5 M | - | + | - | - | - | + | - | - |
| siRNA | Control | | | | Akt | | | |
| COX-2, 72 kDa | | | | | | | | |
| --- | --- | --- | --- | --- | --- | --- | --- | --- |
| COX-1, 72 kDa | | | | | | | | |
| pAkt (Ser473, 60 kDa) | | | | | | | | |
| Akt, 60 kDa | | | | | | | | |
| β-actin, 43 kDa | | | | | | | | |
| IL-4 | - | + | - | + | - | + | - | + |
| IL-1β | - | - | + | + | - | - | + | + |
| siRNA | control | Co-stimulation | | | Akt | | | |
Figure S1. The effects of Akt knockdown on IL-1β-induced COX-2 expression and on the suppressive activity of IL-4.
(A) The effect of Akt knockdown on IL-1β-induced COX-2 expression was analyzed by immunoblotting with FDC-like cells
cultured with the suboptimal doses of IL-1β for 4 h. (B) The effect of Akt knockdown on the suppressive activity of IL-4 was
analyzed by immunoblotting after culturing FDC-like cells with IL-4 (100 U/ml) for 24 h and then with IL-1β (25 pg/ml) for 4 h.
Figure S1

## Slide 2
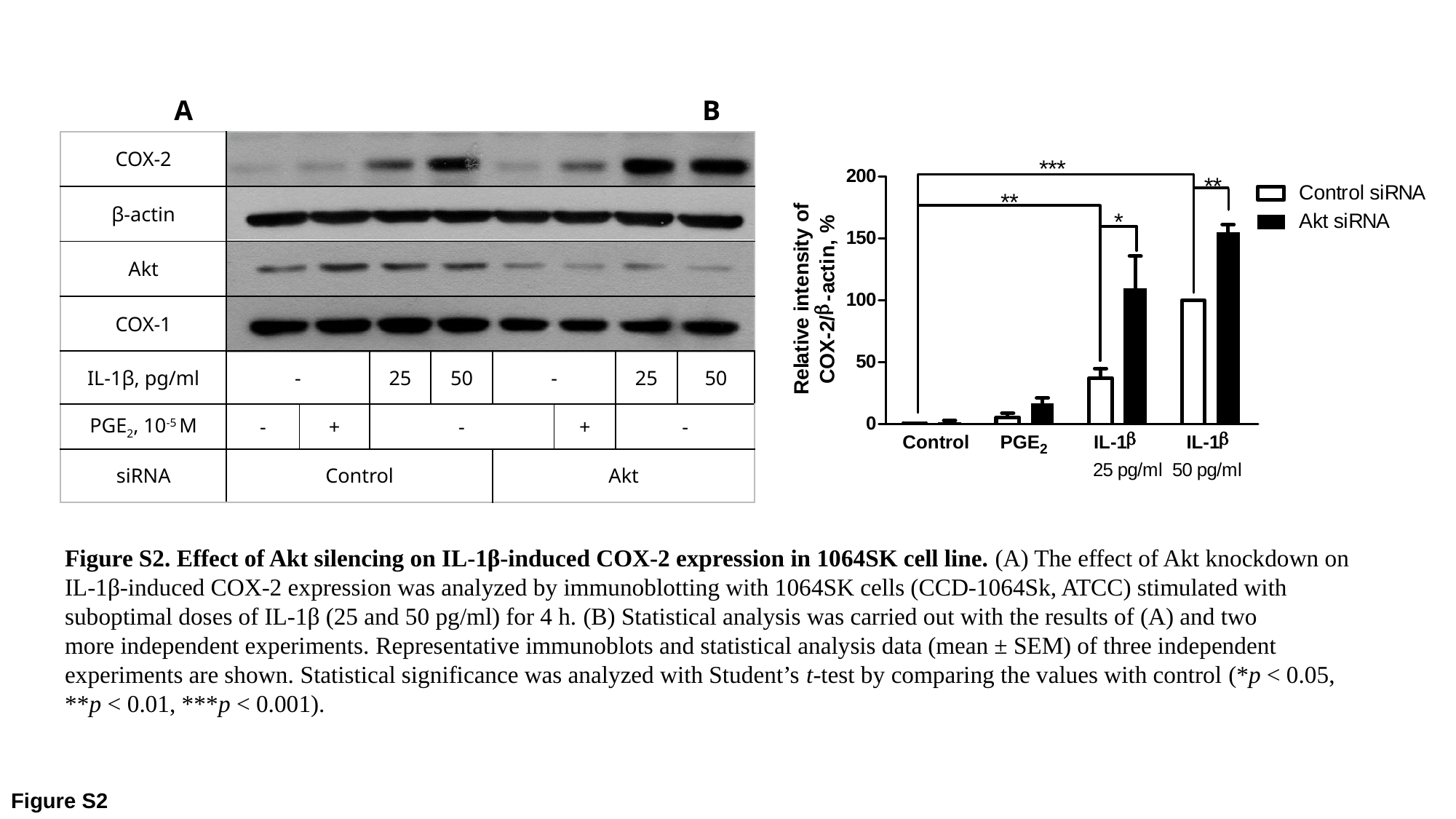

A B
| COX-2 | | | | | | | | |
| --- | --- | --- | --- | --- | --- | --- | --- | --- |
| β-actin | | | | | | | | |
| Akt | | | | | | | | |
| COX-1 | | | | | | | | |
| IL-1β, pg/ml | - | - | 25 | 50 | - | - | 25 | 50 |
| PGE2, 10-5 M | - | + | - | - | - | + | - | - |
| siRNA | Control | | | | Akt | | | |
Figure S2. Effect of Akt silencing on IL-1β-induced COX-2 expression in 1064SK cell line. (A) The effect of Akt knockdown on
IL-1β-induced COX-2 expression was analyzed by immunoblotting with 1064SK cells (CCD-1064Sk, ATCC) stimulated with
suboptimal doses of IL-1β (25 and 50 pg/ml) for 4 h. (B) Statistical analysis was carried out with the results of (A) and two
more independent experiments. Representative immunoblots and statistical analysis data (mean ± SEM) of three independent
experiments are shown. Statistical significance was analyzed with Student’s t-test by comparing the values with control (*p < 0.05,
**p < 0.01, ***p < 0.001).
Figure S2

## Slide 3
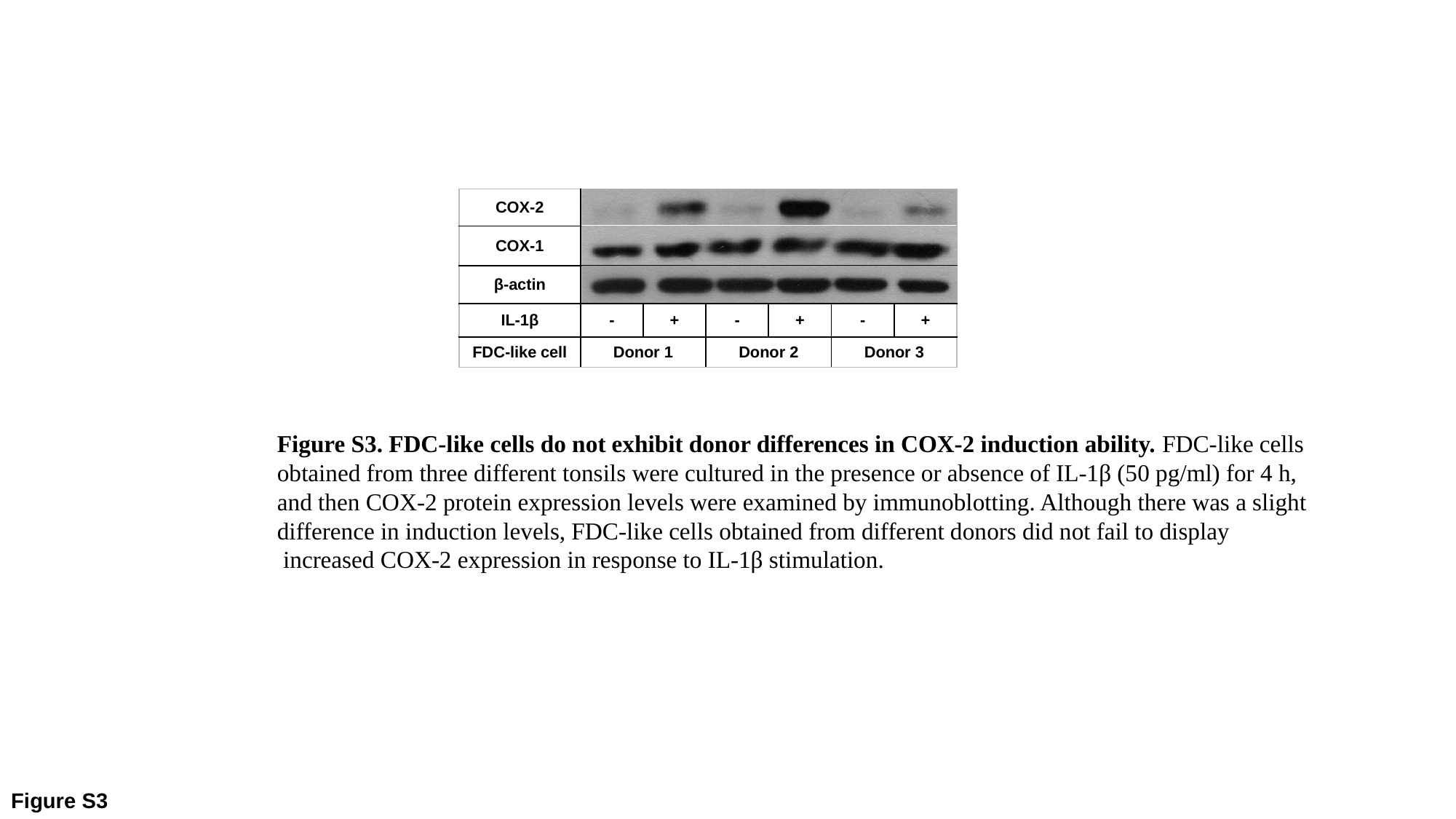

| COX-2 | | | | | | |
| --- | --- | --- | --- | --- | --- | --- |
| COX-1 | | | | | | |
| β-actin | | | | | | |
| IL-1β | - | + | - | + | - | + |
| FDC-like cell | Donor 1 | | Donor 2 | | Donor 3 | |
Figure S3. FDC-like cells do not exhibit donor differences in COX-2 induction ability. FDC-like cells
obtained from three different tonsils were cultured in the presence or absence of IL-1β (50 pg/ml) for 4 h,
and then COX-2 protein expression levels were examined by immunoblotting. Although there was a slight
difference in induction levels, FDC-like cells obtained from different donors did not fail to display
 increased COX-2 expression in response to IL-1β stimulation.
Figure S3
20200610~20200724
